# Digital cognitive phenotyping enhances risk stratification in preclinical Alzheimer's disease

**DOI:** 10.1002/alz.71834

**Published:** 2026-09-15

**Authors:** Casey R. Vanderlip, Craig E. L. Stark

**Affiliations:** ^1^ Department of Neurobiology and Behavior University of California Irvine Irvine California USA

**Keywords:** Alzheimer's disease, blood biomarkers, digital cognitive assessments, early detection, prognosis, risk stratification

## Abstract

**INTRODUCTION:**

Amyloid positivity does not reliably predict near‐term cognitive decline in preclinical Alzheimer's disease (AD). We tested whether digital cognitive phenotyping improves prognostic precision.

**METHODS:**

Baseline performance on a brief self‐administered digital battery was analyzed in 1146 amyloid beta (Aβ)+ and 538 Aβ− cognitively unimpaired older adults from the Anti‐Amyloid Treatment in Asymptomatic Alzheimer's/Longitudinal Evaluation of Amyloid Risk and Neurodegeneration studies. Unsupervised *k*‐means clustering identified cognitive phenotypes. Survival models examined time to Preclinical Alzheimer Cognitive Composite decline and Clinical Dementia Rating (CDR) progression. Associations with tau positron emission tomography and plasma phosphorylated tau (p‐tau)217 were evaluated.

**RESULTS:**

Four cognitive phenotypes emerged. One phenotype (18% of Aβ+ participants) showed the steepest cognitive decline, highest progression on the CDR, greater baseline medial temporal tau, and fastest neocortical tau accumulation over 4.6 years (hazard ratio [HR] for decline ≈ 2.9). Combining this phenotype with elevated plasma p‐tau217 identified a subgroup at markedly amplified risk (HR ≈ 4.5).

**DISCUSSION:**

Digital cognitive phenotyping, especially when integrated with plasma biomarkers, identifies actionable risk heterogeneity and enables scalable precision prognostication in preclinical AD.

## Highlights

- Digital cognitive phenotyping reveals hidden risk in preclinical Alzheimer's disease.
- A distinct subgroup shows accelerated decline and mild cognitive impairment progression.
- The high‐risk phenotype predicts greater baseline and future tau spread.
- Combining plasma phosphorylated tau 217 plus digital profiling markedly enhances prognosis.

## 1 BACKGROUND

Alzheimer's disease (AD) develops slowly, with pathological changes such as amyloid beta (Aβ) accumulation beginning years before the onset of symptoms.^1^ Although Aβ deposition defines the biological presence of AD, it does not directly predict when cognitive decline will begin.^2^ Many individuals with elevated Aβ remain cognitively stable for long periods, while others decline rapidly despite similar biomarker profiles. This unpredictability represents one of the central challenges in clinical practice. Identifying who will decline and when that decline will begin is essential for clinical decision making, patient counseling, and the efficient design of disease‐modifying trials.

The limited ability to estimate the timing of decline has major implications. For patients, a positive biomarker result provides limited guidance about prognosis or near‐term risk. For clinicians, it complicates decisions about when to initiate treatment or increase monitoring as new therapies become available. For researchers and trial sponsors, it makes it difficult to recruit participants who are likely to show measurable change during a study, reducing statistical power and increasing cost. A tool that could identify individuals at imminent risk of cognitive decline would transform both clinical care and therapeutic development in AD.

Cognitive performance provides one of the most direct indicators of emerging impairment. Standardized composites such as the Preclinical Alzheimer Cognitive Composite (PACC) have been instrumental in tracking subtle change and establishing cognitive endpoints for secondary prevention trials.^3^, ^4^ However, because these measures require in‐person administration by trained neuropsychologists in the clinic, they are not readily scalable for widespread use. Detecting meaningful within‐person change, the earliest departure from an individual's own baseline, requires approaches that are both sensitive and scalable across time and populations.^5^

Digital cognitive assessments offer a clinically scalable solution.^6^, ^7^, ^8^ These self‐administered tools can be completed remotely, allowing for frequent and low‐burden testing across large and diverse cohorts. Further, prior work demonstrates that performance on digital cognitive assessments correlate with gold‐standard neuropsychological measures, supporting its validity as a digital measure of cognition in preclinical AD.^9^, ^10^ Beyond accessibility, digital assessments capture detailed performance metrics such as accuracy, reaction time, and variability. These metrics provide additional information, which may capture latent high‐risk behavioral phenotypes that link biological pathology to the earliest signs of functional change.^11^, ^12^

## 2 METHODS

### 2.1 Participants

All data were obtained from the Anti‐Amyloid Treatment in Asymptomatic Alzheimer's (A4) Study, a Phase 3 clinical trial whose design has been described previously.^3^ Briefly, A4 was a double‐blind, placebo‐controlled, 240‐week trial evaluating an anti–Aβ monoclonal antibody in cognitively unimpaired older adults with preclinical AD. A total of 5491 individuals completed screening. Amyloid status was determined using florbetapir positron emission tomography (PET) imaging. Of these, 1146 amyloid‐positive and 538 amyloid‐negative participants were followed longitudinally for up to 4.6 years with repeated cognitive assessments. Amyloid‐positive participants were enrolled in the A4 trial, while amyloid‐negative participants were enrolled in the Longitudinal Evaluation of Amyloid Risk and Neurodegeneration (LEARN) companion study.

### 2.2 Digital cognitive battery

The Computerized Cognitive Composite (C3) is a brief, self‐administered digital cognitive battery completed by all LEARN and A4 participants and described previously.^9^, ^11^, ^13^ The administration time of this battery is 25 to 30 minutes, on average.^11^ It includes six tasks spanning hippocampal memory, attention, processing speed, and working memory: the Mnemonic Similarity Task (MST, née BPS‐O), Face–Name Association Task (FNAME), One Card Learning Task (OCL), Detection Task (DET), Identification Task (IDN), and One‐Back Task (OBT).

In the MST used here, participants encoded 40 everyday objects and later judged 60 test items as “old,” “similar,” or “new.” The primary outcome was the lure discrimination index, taxing pattern separation ability. In FNAME, participants learned 12 face–name pairs and completed recognition and matching trials, with face–name matching accuracy as the primary measure. In OCL, participants viewed a stream of playing cards and indicated whether each had been seen previously, with accuracy as the outcome. DET measured psychomotor speed as the average reaction time across 35 valid trials in which participants responded when a card turned over. IDN assessed visual attention by requiring participants to respond “yes” to red joker cards and “no” to black, with average reaction time as the outcome. OBT measured working memory by asking participants to judge whether each card matched the one immediately before, with average reaction time across 31 trials as the performance metric.

**RESEARCH IN CONTEXT**

1. **Systematic review**: Using PubMed and Google Scholar, we reviewed studies examining amyloid, tau, plasma phosphorylated tau (p‐tau)217, and digital cognitive assessments in preclinical Alzheimer's disease (AD). Prior work has shown that amyloid positivity alone poorly predicts near‐term decline and that tau burden more closely tracks clinical progression. Digital cognitive tools have demonstrated sensitivity to early impairment, but no prior study has used unsupervised digital cognitive phenotyping to identify prognostic subgroups and link them to longitudinal tau accumulation and survival outcomes in a large biomarker‐defined cohort.
2. **Interpretation**: We show that baseline digital cognitive patterns identify a high‐risk phenotype marked by accelerated cognitive decline, elevated tau burden, and faster neocortical tau spread. When combined with plasma p‐tau217, this approach sharply improves near‐term risk stratification in preclinical AD.
3. **Future directions**: Future studies should replicate these findings in more diverse populations, test prospective clinical deployment, and evaluate integration into trial enrichment and treatment decision frameworks.

### 2.3 Neuropsychological assessments

All participants completed in‐person, gold‐standard neuropsychological testing, including the PACC.^4^, ^14^ The PACC comprises four components: the total score on the Free and Cued Selective Reminding Test, delayed paragraph recall from the Logical Memory IIa test, the Digit Symbol Substitution Test, and the Mini‐Mental State Examination total score. To minimize practice effects, alternate versions of these tests were used at each session. To control for baseline performance, each component score was converted to a *z* score by subtracting the baseline mean and dividing by the baseline standard deviation. The PACC score was calculated as the sum of these *z* scores, with negative values indicating decline.

### 2.4 Tau PET

Regional tau PET standardized uptake value ratios (SUVRs) were obtained from A4 imaging and provided from the A4 study team. Similar to prior work, medial temporal lobe tau (MTL tau) was computed as the mean SUVR across bilateral entorhinal and amygdala regions. Neocortical tau was defined as the volume‐weighted average of bilateral inferior and middle temporal regions.^15^

### 2.5 Plasma phosphorylated tau 217

The Lilly Clinical Diagnostics Laboratory measured phosphorylated tau (p‐tau)217 levels using an electrochemiluminescent immunoassay, with sample preparation automated by the Tecan Fluent workstation and detection conducted on the MSD Sector S Imager 600 MM. Values were provided from the A4 study team.

### 2.6 Statistical analyses

All analyses were done in R. Primary clusters were derived using *k*‐means clustering of the 26 digital cognitive assessment measures from the screening visit. Robustness was evaluated by repeating the analyses with percentile‐based splits of composite scores and with hierarchical clustering on the same feature set. To evaluate whether the clustering generalized to unseen participants, we conducted a hold‐out validation analysis within the Aβ+ sample. Specifically, we generated five 70/30 train/test splits (each selected with a random 70% of participants used for training and the other 30% held out for testing). Within each split, *k*‐means clustering (*k* = 4) was fit de novo in the training set using the same digital cognitive features as in the primary analysis. Cluster labels were then aligned to the full‐sample cluster solution, and held‐out participants were assigned to the nearest training‐set centroid.

Survival analyses examined time to cognitive decline on the PACC and time to progression on the Clinical Dementia Rating (CDR). For the PACC, scores were standardized such that all participants began at 0 at baseline, and a decline event was defined as a within‐person decline of at least 0.5 standard deviations, based on the standard deviation from the screening data. An event required that this level of decline be present at two consecutive study visits or at the participant's final observed visit. Similarly, progression on the CDR was defined as two consecutive visits with a non‐zero score or to a final visit with a non‐zero score. Kaplan–Meier curves were generated to visualize survival by amyloid status and by cognitive cluster groups. Cox proportional hazards models were used to estimate hazard ratios, and proportional hazards assumptions were evaluated using Schoenfeld residuals.

Associations between cognitive clusters and Aβ PET SUVR, plasma p‐tau217, and tau PET were examined with linear mixed effects models with random intercepts for participant. Fixed effects included cluster, region (medial temporal vs. neocortical), time, and their interactions. Estimated marginal means and Tukey‐adjusted comparisons were used to compare clusters within each region.

To evaluate the combined effects of plasma p‐tau217 and cognitive profiles, we conducted additional survival analyses incorporating high p‐tau217, defined as values in the upper tertile (≥ 0.28 pg/mL), consistent with prior work identifying this threshold as high risk.^16^ Two types of Cox models were estimated. First, mutually exclusive groups were constructed by categorizing participants into non‐overlapping combinations of amyloid status, high risk cluster membership, and high p‐tau217, hazard ratios from these models reflect the risk associated with each specific group relative to Aβ– individuals. Second, non–mutually exclusive models were fit in which high risk cluster membership and high p‐tau217 were entered simultaneously as independent predictors, allowing estimation of their separate and additive effects on risk.

## 3 RESULTS

### 3.1 Most individuals with preclinical AD do not decline over 4.6 years

**FIGURE 1:**
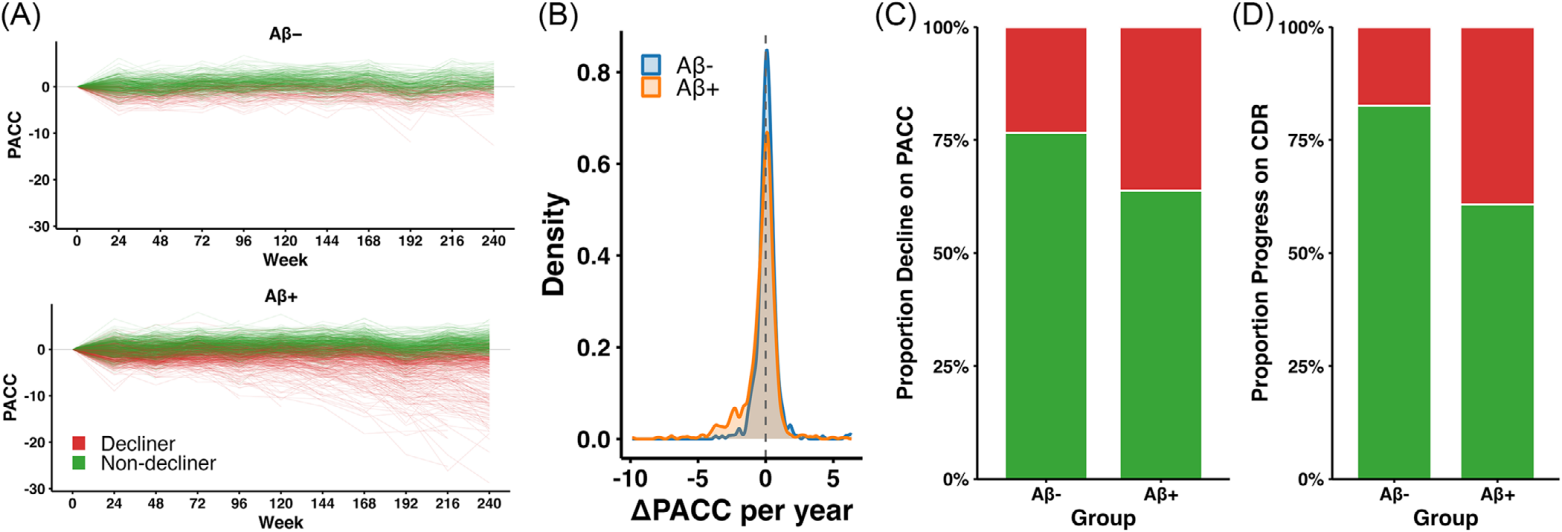
Cognitive trajectories and rates of decline in Aβ– and Aβ+ individuals. A, Individual PACC trajectories over 240 weeks for Aβ– (top) and Aβ+ (bottom) participants. Although group means remain near baseline, a subset of individuals in each group shows meaningful decline (red). B, Density distributions of annualized PACC change demonstrate substantial overlap between Aβ– and Aβ+ individuals. C, Proportion of participants who decline on the PACC, showing a higher rate of decline among Aβ+ individuals. D, Proportion of participants progressing to cognitive impairment, again demonstrating increased progression in the Aβ+ group. Together, these panels illustrate that while Aβ+ positivity increases overall risk, most individuals remain stable and amyloid alone provides limited prognostic precision. Aβ, amyloid beta; CDR, Clinical Dementia Rating; PACC, Preclinical Alzheimer Cognitive Composite.

Cognitive performance remained largely stable over the 4.6‐year follow‐up, with only a subset of participants showing decline on the PACC (Figure 1A). The distribution of annualized PACC change overlapped substantially between Aβ– and Aβ+ participants (Figure 1B), indicating that amyloid positivity alone was not sufficient to distinguish individuals who would experience cognitive decline. Aβ+ participants were significantly more likely to show cognitive decline on the PACC and exhibit clinical progression on the CDR. On the PACC, 36.2% of Aβ+ participants declined compared to 23.4% of Aβ– participants (odds ratio [OR] = 1.85, 95% confidence interval [CI] 1.46–2.36; Figure 1C). Similarly, 39.3% of Aβ+ participants progressed on the CDR compared to 17.4% of Aβ– participants (OR = 3.08, 95% CI 2.34–4.07; Figure 1D). Despite these differences, many participants in both groups remained cognitively stable over 4.6 years, underscoring that amyloid positivity is not sufficient on its own to predict imminent decline.

### 3.2 Clustering on performance on a digital cognitive assessment identifies a subgroup at high risk for future decline

**FIGURE 2:**
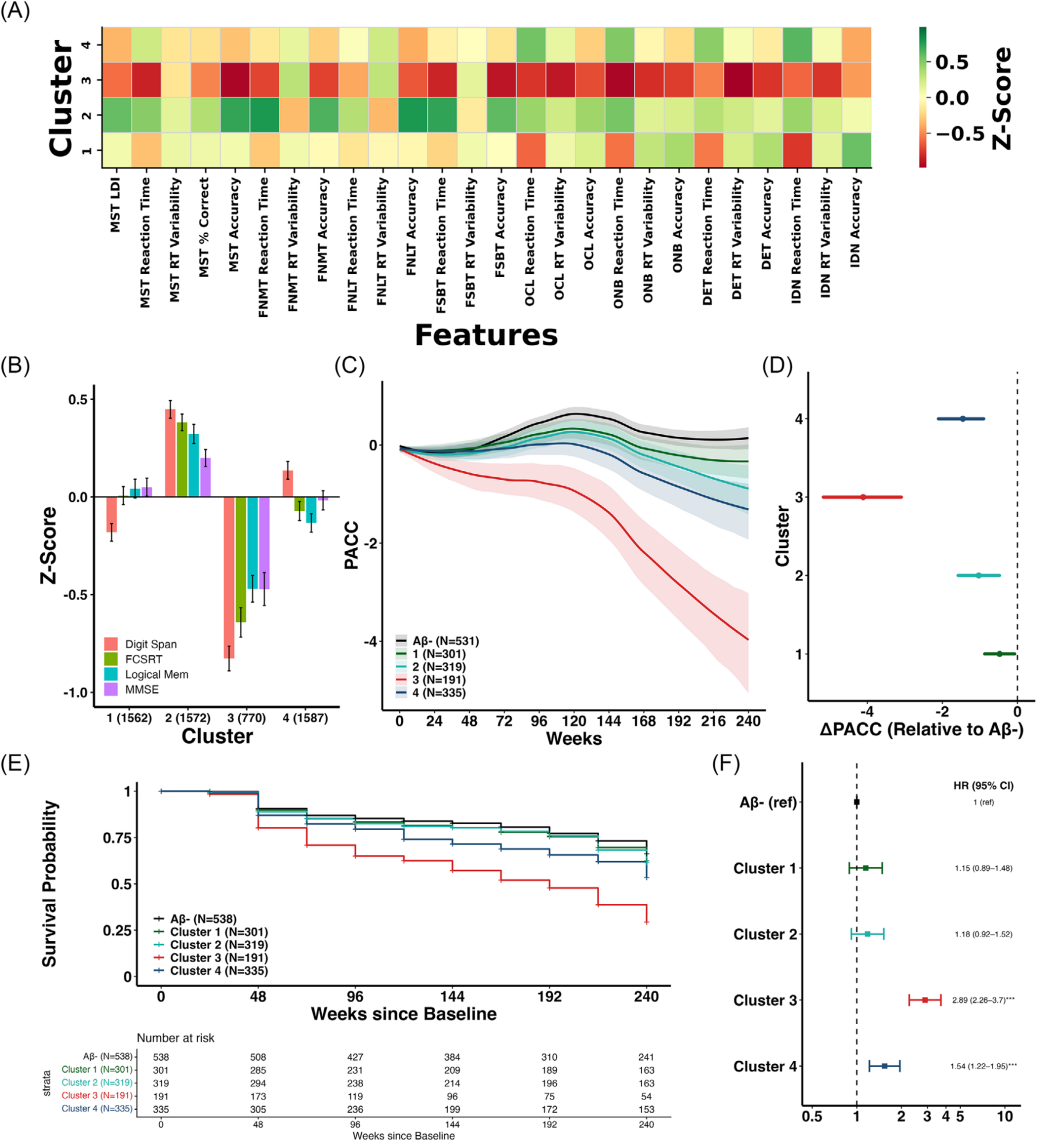
Digital cognitive phenotypes and their links to baseline cognition and long‐term decline. A, Heatmap of standardized digital task features showing four distinct cognitive clusters derived from *k*‐means clustering in amyloid‐positive individuals. B, Baseline neuropsychological performance across clusters, with Cluster 3 showing broad deficits and Cluster 2 showing above‐average performance. C, Estimated 240‐week PACC trajectories demonstrating markedly accelerated decline in Cluster 3 compared to other clusters and Aβ– participants. D, Annualized PACC change relative to Aβ– participants, highlighting large deficits in Cluster 3 and modest decline in Cluster 4. E, Kaplan–Meier survival curves for time to meaningful PACC decline, revealing substantially higher progression risk in Cluster 3. F, Hazard ratios (95% CI) confirming that Cluster 3 has the highest risk of decline, followed by Cluster 4, while Clusters 1 and 2 show no elevated risk. These results indicate that a single digital cognitive assessment identifies cognitive subgroups with distinct neuropsychological profiles and markedly different trajectories of decline. Aβ, amyloid beta; CI, confidence interval; DET, Detection Task; FSBT, face recognition; FNLT, first letter name recall; FNMT, face‐name matching; HR, hazard ratio; IDN, Identification Task; LDI, lure Discrimination Index; MST, Mnemonic Similarity Task; OCL, One Card Learning Task; ONB, One‐Back Task; PACC, Preclinical Alzheimer Cognitive Composite; RT, reaction time.

To identify distinct cognitive phenotypes within Aβ+ individuals, we applied unsupervised *k*‐means clustering (*k* = 4) to baseline performance across all six digital tasks. We evaluated the stability and separation of this clustering solution using bootstrap resampling and silhouette analyses. Silhouette analyses across candidate values of *k* showed that *k* = 2 provided the strongest coarse separation, but the four‐cluster solution was retained because it provided greater clinical resolution and separated a reproducible high‐risk subgroup (Figure S1A in supporting information). Bootstrap analyses further indicated that the four‐cluster solution was stable, including the high‐risk Cluster 3 phenotype (all clusters Jaccard values > 0.9; Figure S1B). The four clusters differed in performance on the C3, characterized by distinct patterns of accuracy, reaction time, and variability (Figure 2A and Table S1 in supporting information). Cluster 1 showed near‐average performance across measures, Cluster 2 demonstrated slightly faster reaction times with modest accuracy reductions, Cluster 3 exhibited broad deficits across metrics; and Cluster 4 showed slightly below‐average accuracy and speed. Baseline neuropsychological performance on the PACC and its subtests mirrored these cognitive profiles (Figure 2B; Figure S2A in supporting information and Table S1). Cluster 1 showed average performance with subtle deficits on Digit Span, Cluster 2 performed above average across all measures, Cluster 3 exhibited deficits across all tests, and Cluster 4 showed generally average performance with mild deficits on memory‐based assessments.

There were notable differences across baseline demographic and clinical features across the cognitive clusters (Table 1). The clusters were broadly similar in education and apolipoprotein E (*APOE*) genotype distribution, suggesting that the high‐risk phenotype was not simply explained by lower education or higher *APOE* ε4 carriership. However, Cluster 3 was somewhat older, had a lower proportion of female participants, and showed higher baseline amyloid burden.

**TABLE 1:** Demographics Table.

Cluster	1	2	3	4
*N*	1562	1572	770	1587
Age, mean (SD)	71.2 (4.4)	69.7 (3.9)	74.9 (5.4)	71.7 (4.7)
Education, mean (SD)	16.5 (2.9)	16.9 (2.7)	16.1 (3.2)	16.5 (2.9)
Female, *n* (%)	947 (60.6%)	1064 (67.7%)	427 (55.5%)	791 (49.8%)
Amyloid Centiloid, mean (SD)	19.8 (31.6)	20.3 (31.7)	36.9 (46.9)	22.5 (33.7)
*APOE* genotype				
ε2/ε2, n (%)	8 (0.5%)	5 (0.3%)	6 (0.8%)	10 (0.7%)
ε2/ε3, n (%)	170 (11.3%)	148 (9.7%)	65 (9.1%)	158 (10.3%)
ε3/ε3, n (%)	835 (55.7%)	799 (52.6%)	382 (53.7%)	852 (55.8%)
ε2/ε4, n (%)	32 (2.1%)	32 (2.1%)	23 (3.2%)	41 (2.7%)
ε3/ ε4, n (%)	416 (27.7%)	484 (31.8%)	213 (29.9%)	419 (27.4%)
ε4/ε4, n (%)	39 (2.6%)	52 (3.4%)	23 (3.2%)	48 (3.1%)
Race				
American Indian or Alaskan Native, *n* (%)	3 (0.2%)	2 (0.1%)	2 (0.3%)	4 (0.3%)
Asian, *n* (%)	62 (4.0%)	19 (1.2%)	18 (2.3%)	95 (6.0%)
Black or African American, *n* (%)	80 (5.1%)	25 (1.6%)	55 (7.1%)	68 (4.3%)
More than one race, *n* (%)	9 (0.6%)	11 (0.7%)	14 (1.8%)	6 (0.4%)
Native Hawaiian or other Pacific Islander, *n* (%)	1 (0.1%)	NA	NA	2 (0.1%)
Unknown or not reported, *n* (%)	9 (0.6%)	9 (0.6%)	12 (1.6%)	8 (0.5%)
White, *n* (%)	1398 (89.5%)	1506 (95.8%)	669 (86.9%)	1404 (88.5%)
MMSE, mean (SD)	28.8 (1.2)	29.0 (1.1)	28.1 (1.5)	28.7 (1.3)

Abbreviations: *APOE*, apolipoprotein E; MMSE, Mini‐Mental State Examination; SD, standard deviation.

We asked whether cluster membership, determined from baseline cognitive performance, conferred information concerning longitudinal cognitive change (Figure 2C and Figure S2B–E). Relative to Aβ– participants, mean 240‐week within‐person PACC change was –0.47 (95% CI = −0.87 to −0.07) for Cluster 1, −1.03 (−1.58 to −0.48) for Cluster 2, −4.11 (−5.17 to −3.09) for Cluster 3, and −1.45 (−2.11 to −0.90) for Cluster 4 (Figure 2D). Strikingly, Cluster 3 declined nearly four points more than Aβ– participants, indicating a markedly accelerated within‐person trajectory.

To evaluate the robustness of these findings and whether they depended on the number of clusters, we compared the observed cluster solution with alternative data‐partitioning methods (Figure S3 in supporting information). Far less robust effects were observed when participants were divided by percentile splits on overall composite measures, regardless of the number of splits (Figure S3A–D); the lowest performing percentile declined only marginally more than other groups. In contrast, both *k*‐means (Figure S3E–H) and hierarchical clustering (Figure S3J–M), across a range of cluster numbers, consistently identified a distinct subgroup that declined substantially more than others. Further, to assess whether the cluster solution generalized to unseen participants, we performed a hold‐out validation analysis using five independent 70/30 train/test splits of the Aβ+ sample (Figure S4A–E in supporting information). Across splits, the held‐out clusters reproduced the same broad feature structure observed in the primary analysis, including a distinct Cluster 3 pattern marked by relatively broad impairment across digital cognitive measures. Importantly, held‐out participants assigned to Cluster 3 consistently showed the steepest longitudinal PACC decline, whereas held‐out participants assigned to Clusters 1, 2, and 4 showed substantially less decline. These findings suggest that the cognitive phenotypes, particularly the high‐risk Cluster 3 profile, were not specific to the original sample used to derive the clusters and can be recovered in unseen participants.

We next asked whether individuals in different clusters reached meaningful decline at different rates. Survival analyses of PACC decline showed the highest risk in Cluster 3 (hazard ratio [HR] = 2.89, 95% CI 2.26–3.77, *p* < 0.001), consistent with its pronounced decline (Figure 2E–F). Cluster 4 also showed elevated risk (HR = 1.54, 95% CI 1.24–1.95), reflecting modestly greater longitudinal decline relative to Aβ– participants. Cluster 1 (HR = 1.15, 95% CI 0.89–1.48) and Cluster 2 (HR = 1.19, 95% CI 0.92–1.52) did not differ significantly from the Aβ– reference group. Importantly, nearly identical results were seen when looking at CDR progression (Figure S5 in supporting information).

To determine whether these associations were robust to baseline covariates, we repeated the survival analyses adjusting for age, sex, education, *APOE* ε4 carriership, and treatment assignment. The results were nearly identical. For PACC decline, Cluster 3 remained significantly associated with elevated risk after adjustment (Figure S6A–B in supporting information, HR = 2.01, 95% CI = 1.51–2.68, *p* < 0.001), whereas Cluster 1 (HR = 0.95, 95% CI = 0.72–1.26), Cluster 2 (HR = 1.00, 95% CI = 0.75–1.32), and Cluster 4 (HR = 1.22, 95% CI = 0.93–1.59) were not significantly associated with PACC decline. For CDR progression, Cluster 3 again showed the highest risk after adjustment (Figure S6C–D; HR = 3.27, 95% CI = 2.34–4.56, *p* < 0.001), with elevated risk also observed for Cluster 1 (HR = 1.42, 95% CI = 1.02–1.98) and Cluster 4 (HR = 1.91, 95% CI = 1.39–2.63). Together, this suggests that patterns of performance on the digital assessment predict which individuals are at highest risk for meaningful decline.

### 3.3 Baseline and longitudinal AD biomarker burden differ across cognitive subtypes

**FIGURE 3:**
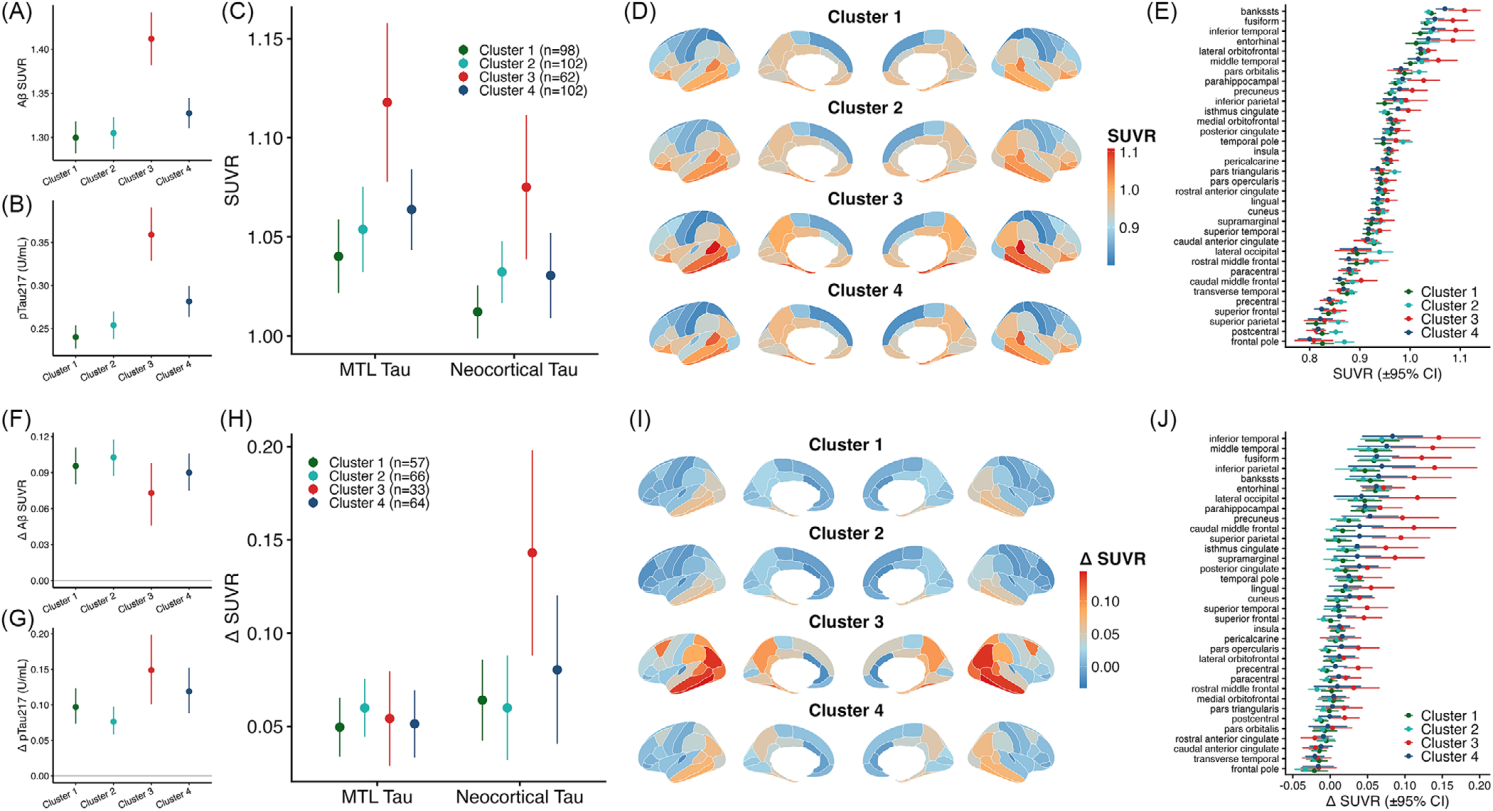
Baseline and longitudinal AD biomarker burden differ across digital cognitive phenotypes. A, Baseline Aβ PET SUVR across clusters, showing significantly higher amyloid burden in Cluster 3. B, Baseline plasma p‐tau217 levels across clusters, showing higher p‐tau217 in Cluster 3. C, Baseline MTL and neocortical tau SUVRs across clusters. D, Cortical maps of baseline tau burden by cluster. E, Regional baseline tau SUVR estimates with 95% confidence intervals, showing elevated tau burden in Cluster 3 across temporal and association cortical regions. F, Longitudinal change in Aβ PET SUVR, showing no significant cluster differences in amyloid accumulation over follow‐up. G, Longitudinal change in plasma p‐tau217, showing significant cluster differences in p‐tau217 change. H, Longitudinal change in MTL and neocortical tau SUVRs, showing selectively greater neocortical tau accumulation in Cluster 3. I, Cortical maps of longitudinal tau change by cluster. J, Regional ΔSUVR estimates with 95% confidence intervals, confirming greater neocortical tau accumulation in Cluster 3. Together, these results indicate that the high‐risk digital cognitive phenotype is associated with greater baseline AD biomarker burden and downstream tau‐related disease activity, but not faster amyloid accumulation over follow‐up. AD, Alzheimer's disease; CI, confidence interval; MTL, medial temporal lobe; PET, positron emission tomography; p‐tau, phosphorylated tau; SUVR, standardized uptake value ratio.

We next examined whether cognitive clusters differed in baseline AD biomarker burden and longitudinal biomarker change. At baseline, clusters differed significantly in Aβ PET SUVR (Figure 3A, *F*[31142] = 19.22, *p* < 0.001) and plasma p‐tau217 (Figure 3B, *F*[31057] = 24.54, *p* < 0.001). Tukey‐adjusted post hoc comparisons confirmed that Cluster 3 had significantly higher baseline Aβ SUVR and p‐tau217 than all other clusters (*p*s < 0.001).

Next, in the subset of participants with tau PET, we examined whether participants across different clusters differed in baseline tau burden. There were main effects of cluster (Figure 3C, Figure S7 in supporting information, *F*[3358.7] = 7.05, *p* < 0.001) and region (MTL vs. neocortical; *F*[1357.6] = 31.64, *p* < 0.001), with no interaction (*F*[3357.6] = 0.62, *p* = 0.604). Estimated marginal means showed higher MTL tau in Cluster 3 compared to Cluster 1 (*p* = 0.0001), Cluster 2 (*p* = 0.0015), and Cluster 4 (*p* = 0.0106); other MTL pairwise differences were not reliable. For neocortical tau, Cluster 3 exceeded Cluster 1 (*p* = 0.0028) and showed modest evidence for exceeding Cluster 2 (*p* = 0.068) and Cluster 4 (*p* = 0.054), suggesting that Cluster 3 has the highest baseline tau burden, particularly in medial temporal regions. Similar results were present when doing a regional‐based analysis (Figure 3D–E).

We next examined longitudinal biomarker change. Clusters did not differ significantly in longitudinal Aβ accumulation (Figure 3F, ΔAβ SUVR: *F*[3797] = 1.71, *p* = 0.164). In contrast, clusters differed significantly in longitudinal plasma p‐tau217 change (Figure 3G, Δp‐tau217: *F*[3650] = 3.56, *p* = 0.014) with Cluster 3 having significantly greater p‐tau217 increase than Cluster 2 (*p* = 0.013), although differences from Cluster 1 (difference = 0.052, *p* = 0.134) and Cluster 4 (difference = 0.030, *p* = 0.595) were not reliable.

We next tested whether clusters differed in future tau accumulation over the 4.6‐year follow‐up. There was a main effect of region (MTL vs. neocortical; Figure 3H, Figure S7, *F*[1215.6] = 15.83, *p* < 0.001) and a significant interaction (F[3215.7] = 4.37, *p* = 0.005), but no main effect of cluster (F[3216.4] = 1.90, *p* = 0.130). Follow‐up comparisons revealed that differences were confined to neocortical tau. Cluster 3 accumulated more neocortical tau than Cluster 1 (*p* = 0.004), Cluster 2 (*p* = 0.001), and Cluster 4 (*p* = 0.027), whereas no other groups differed (all *p*s > 0.694). Further, no differences were observed for MTL tau (all *p* > 0.900). Again, regional analyses confirmed these findings (Figure 3I–J). These findings indicate that Cluster 3 exhibited selectively greater neocortical tau accumulation over 4.6 years.

To further evaluate whether Cluster 3 was enriched for individuals who had reached elevated tau burden at baseline, we examined the proportion of participants exceeding multiple SUVR thresholds for MTL and neocortical tau (Figure S8 in supporting information). Across thresholds, Cluster 3 consistently showed the highest proportion of tau‐positive individuals. For MTL tau, 45.2% of Cluster 3 exceeded SUVR ≥ 1.1, compared to 20.4% of Cluster 1, 23.5% of Cluster 2, and 27.5% of Cluster 4. This pattern remained at higher thresholds: 22.6% of Cluster 3 exceeded SUVR ≥ 1.2 compared to 7.1%, 8.8%, and 10.8% of Clusters 1, 2, and 4, respectively, and 11.3% of Cluster 3 exceeded SUVR ≥ 1.3 compared to 1.0%, 4.9%, and 4.9% of Clusters 1, 2, and 4, respectively. A similar pattern was observed for neocortical tau. At SUVR ≥ 1.1, 27.4% of Cluster 3 was neocortical tau+, compared to 13.4%, 15.7%, and 13.9% of Clusters 1, 2, and 4, respectively. At SUVR ≥ 1.2, 16.1% of Cluster 3 was neocortical tau+ compared to 1.0%, 1.0%, and 6.9% of Clusters 1, 2, and 4, respectively.

### 3.4 Accelerated cognitive decline in individuals with both high p‐tau217 and at‐risk cognitive phenotype

**FIGURE 4:**
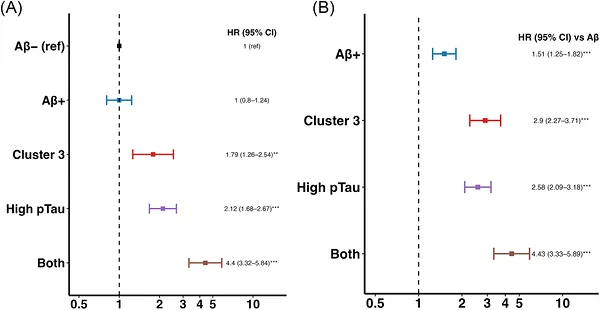
Additive effects of cognitive vulnerability and p‐tau217 on risk of decline. A, Hazard ratios for cognitive decline are shown for mutually exclusive groups defined by amyloid status, Cluster 3 membership, elevated p‐tau217, and the combination of both high‐risk features, referenced to Aβ– participants. B, Non–mutually exclusive models demonstrate that both Cluster 3 membership and elevated p‐tau217 independently contribute to increased risk, and individuals with both factors show the greatest overall risk. Error bars represent 95% confidence intervals. Aβ, amyloid beta; CI, confidence interval; HR, hazard ratio; p‐tau, phosphorylated tau.

To determine whether digital cognitive assessments and plasma p‐tau217 provide complementary prognostic information, we next examined their independent and combined associations with risk of decline. Both factors were individually associated with significantly higher risk, and this risk increased even further when both were present. In mutually exclusive models, referenced to Aβ– participants, individuals who were neither in Cluster 3 nor had high p‐tau217 did not decline at a different rate than Aβ– participants (Figure 4A; HR = 1.00, 95% CI = 0.80–1.24). Membership in Cluster 3 conferred an increased risk (HR = 1.79, 95% CI = 1.26–2.54), as did high p‐tau217 alone (HR = 2.12, 95% CI = 1.68–2.67). However, individuals exhibiting both Cluster 3 cognitive performance and elevated p‐tau217 had markedly greater risk than either factor individually (HR = 4.40, 95% CI = 3.32–5.84), reflecting an amplified effect. This pattern held in non–mutually exclusive models using Aβ– as the reference group: Cluster 3 alone (Figure 4B; HR = 2.90, 95% CI = 2.27–3.71) and high p‐tau217 alone (HR = 2.58, 95% CI = 2.09–3.18) each significantly increased risk, but the combination again produced the highest HR (HR = 4.49, 95% CI = 3.33–5.89). Together, these findings indicate that although each marker identifies individuals at elevated risk, their co‐occurrence identifies a subgroup with an especially pronounced likelihood of imminent decline.

## 4 DISCUSSION

Predicting which individuals with preclinical AD will decline in the near term remains a central unmet need in AD research and clinical care. Aβ identifies biological AD, yet individuals with similar amyloid burden often show strikingly different cognitive and pathological trajectories. Our findings demonstrate that a low‐burden digital cognitive assessment can reveal a high‐risk cognitive phenotype characterized by substantially greater risk of meaningful cognitive decline, progression on the CDR, elevated baseline tau, and accelerated future tau spread. When paired with plasma p‐tau217, this approach provides even greater prognostic precision, highlighting that digital cognitive signatures reflect early functional vulnerability and offer a feasible pathway toward individualized risk assessment in preclinical AD.

### 4.1 A cluster‐based digital cognitive signature predicts AD progression

By applying unsupervised clustering to performance across a digital cognitive battery, we identified distinct cognitive phenotypes within Aβ+ older adults. Among these, one subgroup, marked by subtle impairments on the digital cognitive assessment, declined nearly three times more on the PACC than Aβ– participants and were substantially more likely to reach meaningful decline or progress to mild cognitive impairment (CDR progression) despite all participants being cognitively unimpaired at baseline. Importantly, this high risk phenotype was not detectable using traditional percentile‐based approaches. Digital performance profiles captured latent patterns that carried strong prognostic value, emphasizing that the structure, not just the magnitude, of performance contains early markers of vulnerability.

A striking and important finding was the alignment between the cognitive phenotype and tau pathology. Consistent with growing evidence that tau, rather than amyloid, drives cognitive change, individuals in the vulnerable cluster showed significantly higher MTL tau at baseline and, critically, the greatest neocortical tau accumulation over 4.6 years. This pattern is consistent with models of tau progression in preclinical AD, in which medial temporal tau reflects early disease involvement and neocortical tau accumulation marks a more advanced and clinically relevant phase of disease spread.^15^, ^17^, ^18^, ^19^, ^20^ The enrichment of Cluster 3 for elevated baseline MTL tau, combined with its greater subsequent neocortical tau accumulation, suggests that this high‐risk cognitive phenotype may capture individuals undergoing active cortical tau propagation. This may help explain why this subgroup showed elevated risk for near‐term decline.

More broadly, this subgroup may provide a useful platform for developing more comprehensive prognostic models that integrate cognition, plasma biomarkers, imaging, genetics, and markers of co‐pathology. While Cluster 3 showed greater baseline amyloid and p‐tau217 levels, this group did not show faster longitudinal amyloid accumulation, suggesting that its elevated risk was not driven simply by continued amyloid deposition. Increased tau accumulation, conversely, likely accounts for part of the elevated risk observed in Cluster 3, but the biological basis of this vulnerability remains incompletely understood and may also involve altered hippocampal function, neuroinflammation, hyperexcitability, vascular injury, or other co‐pathologies such as α‐synuclein.^21^, ^22^, ^23^, ^24^, ^25^

The importance of identifying individuals at risk of accelerated cognitive decline and tau accumulation is critical. Tau‐targeted therapeutics are advancing rapidly, and clinical trials increasingly require biomarkers that predict near‐term change.^26^, ^27^ Moreover, as therapies enter routine care, clinicians will need tools that determine which individuals warrant treatment initiation, closer monitoring, or further evaluation. Our results point to a scalable remote digital assessment to refine risk estimation.^6^, ^28^ Such an approach could help clinicians prioritize individuals most in need of intervention while reducing unnecessary imaging or specialist referral for those at low risk of near‐term progression.

### 4.2 Integrating a cluster‐based digital cognitive signature with plasma biomarkers aids in risk stratification

Plasma p‐tau217 is emerging as one of the most accurate and promising blood‐based indicators of AD pathology, with growing integration into diagnostic workflows and increasing relevance for both therapeutic eligibility and prognosis.^29^, ^30^ Yet, similar to amyloid, elevated p‐tau217 alone may not fully explain the heterogeneity in clinical trajectories among cognitively unimpaired individuals. Our findings demonstrate that individuals who not only exhibit elevated plasma p‐tau217 but also fall into the high‐risk digital cognitive group face a markedly greater likelihood of cognitive decline than those identified by either factor alone. This combined effect indicates that these markers capture distinct but converging pathways of disease progression. The ability to detect this using a brief assessment and a blood draw highlights a powerful, low‐burden approach for stratifying risk in preclinical AD.^13^, ^31^ In clinical practice, such a strategy could help identify individuals who merit closer monitoring and earlier follow‐up imaging, or are candidates for emerging therapeutics, while sparing low‐risk individuals from unnecessary procedures.

### 4.3 Implications and limitations

These findings support a potential clinical pathway for identifying risk in cognitively unimpaired adults. A brief digital assessment could serve as a scalable first step in widespread screening, allowing large populations to be evaluated without imaging, invasive testing, or specialist appointments. This makes it possible to direct biomarker confirmation and closer follow‐up to the individuals who show the earliest signs of vulnerability, rather than applying resource‐intensive testing to everyone. As disease‐modifying therapies (e.g., donanemab) are now approved for symptomatic patients and demonstrate their greatest benefit when initiated earlier in the disease course, the ability to recognize elevated risk before symptoms emerge is essential.^27^ Combined with blood‐based markers like p‐tau217, digital tools are a clinically scalable strategy for pinpointing individuals approaching symptom onset and guiding timely intervention in AD.

Several limitations warrant consideration. The A4/LEARN cohort is highly educated, and replication in more diverse samples is needed to ensure generalizability. Additionally, although we examined tau PET and p‐tau217, other biomarkers may further refine prediction. Nonetheless, the current findings provide a foundation for integrating digital and biomarker measures for prognosis.

### 4.4 Conclusion

In sum, our study demonstrates that a digital cognitive assessment can identify latent behavioral phenotypes that predict accelerated tau spread and near‐term cognitive decline in preclinical AD. When combined with plasma pT‐tau217, this approach pinpoints a subgroup at exceptionally high risk, precisely the individuals in whom early intervention, closer monitoring, and tau‐targeted therapies are likely to have the greatest impact. These findings offer a practical path toward precision prognostication in AD.

## CONFLICT OF INTEREST STATEMENT

The authors declare no conflicts of interest. Author disclosures are available in the Supporting Information.

## CONSENT STATEMENT

All subjects provided written informed consent.

## Supporting information



Supporting Information

Supporting Information
