# Supplementary material for: Digital cognitive phenotyping enhances risk stratification in preclinical Alzheimer's disease
Source: Alzheimers Dement. 2026 Sep 15;22(9):e71834. doi: 10.1002/alz.71834 (PMC13575981; doi:10.1002/alz.71834)
Supplement: Supplementary file 2 [file ALZ-22-e71834-s002.docx]

Supplemental

Supplemental Table 1

| Measure | Cluster 1 N; mean (SD) | Cluster 2 N; mean (SD) | Cluster 3 N; mean (SD) | Cluster 4 N; mean (SD) |
| --- | --- | --- | --- | --- |
| C3 composite | N=1527; -0.30 (0.39) | N=1571; 0.23 (0.36) | N=769; -0.85 (0.49) | N=1517; -0.19 (0.40) |
| Learning/working memory composite | N=1557; 0.13 (0.69) | N=1573; 0.15 (0.70) | N=768; -0.92 (0.87) | N=1577; -0.42 (0.75) |
| Psychomotor/attention composite | N=1567; -0.72 (0.69) | N=1573; 0.31 (0.65) | N=768; -0.63 (0.91) | N=1585; 0.48 (0.55) |
| Attention composite | N=1558; -0.74 (0.58) | N=1573; 0.33 (0.61) | N=766; -0.81 (0.80) | N=1578; 0.44 (0.52) |
| Detection | N=1568; 2.66 (0.10) | N=1573; 2.57 (0.08) | N=770; 2.66 (0.12) | N=1587; 2.56 (0.08) |
| Identification | N=1567; 2.84 (0.07) | N=1573; 2.74 (0.06) | N=771; 2.83 (0.09) | N=1585; 2.72 (0.05) |
| One Card Learning | N=1565; 55.23 (7.61) | N=1573; 57.33 (7.47) | N=773; 48.68 (7.85) | N=1585; 51.89 (8.02) |
| One Back | N=1559; 31.09 (0.84) | N=1573; 31.20 (0.67) | N=772; 31.44 (2.35) | N=1579; 31.33 (1.08) |
| Face Name Memory Task | N=1521; 8.06 (1.75) | N=1570; 9.31 (1.54) | N=765; 6.56 (2.02) | N=1508; 7.58 (1.76) |
| Behavioral Pattern Separation Test | N=1532; 0.41 (0.18) | N=1574; 0.52 (0.17) | N=773; 0.28 (0.20) | N=1532; 0.32 (0.19) |
| PACC composite | N=1564; -1.08 (2.12) | N=1573; 0.26 (2.07) | N=772; -3.29 (2.60) | N=1590; -1.10 (2.24) |
| FCSRT free and cued total recall | N=1562; 76.14 (5.66) | N=1572; 78.41 (5.25) | N=770; 72.21 (6.48) | N=1589; 75.66 (5.94) |
| Logical Memory delayed recall | N=1564; 11.81 (3.26) | N=1575; 12.74 (3.32) | N=772; 10.07 (3.26) | N=1592; 11.21 (3.20) |
| Digit Symbol total score | N=1564; 41.71 (8.27) | N=1573; 47.46 (8.36) | N=772; 35.80 (8.16) | N=1590; 44.60 (8.43) |
| MMSE | N=1564; 28.81 (1.18) | N=1573; 29.00 (1.12) | N=772; 28.15 (1.52) | N=1588; 28.72 (1.27) |

Supplementary Figure 1Cluster selection and stability analyses. (A) Average silhouette scores across candidate k-means solutions from k = 2 to k = 8. The dashed line indicates the four-cluster solution used in the primary analyses. Although k = 2 showed the highest silhouette score, k = 4 was retained because it provided greater clinical resolution while preserving the high-risk cognitive phenotype. (B) Bootstrap stability of the four-cluster solution, shown as cluster-wise Jaccard similarity across resampled datasets. Dashed and dotted horizontal lines indicate commonly used thresholds for stable and moderately stable clusters, respectively. All four clusters showed high bootstrap stability.

Supplementary Figure 2. Baseline PACC performance and longitudinal PACC trajectories across digital cognitive clusters. (A) Distribution of screening PACC scores by cluster. Points represent individual participants, with violin plots showing the cluster-level distribution. (B–E) Individual within-person PACC trajectories from baseline through 240 weeks for Clusters 1–4. Cluster 3 showed lower baseline PACC performance and greater heterogeneity in longitudinal decline, including a subset of participants with pronounced decline over follow-up.

Supplementary Figure 3. Longitudinal PACC trajectories across alternative phenotyping approaches. A–D) Percentile-based grouping of digital task performance using A) three, B) four, C) five, D) or six groups. E–H) Phenotypes derived from k-means clustering using four to six cluster solutions. I–L) Phenotypes derived from hierarchical clustering using four to six clusters. In all panels, solid lines depict estimated mean PACC trajectories over 240 weeks and shaded regions represent bootstrap-derived 95 percent confidence intervals. The Aβ- reference group is shown in black, with phenotype-specific trajectories among Aβ+ individuals shown in color. Across both k-means and hierarchical clustering approaches, a single subgroup consistently exhibits substantially faster decline, demonstrating that the high-risk phenotype observed in the primary analysis is robust across multiple methods.

Supplementary Figure 4. Hold-out validation of digital cognitive phenotypes across five independent 70/30 train/test splits. For each split, k-means clustering was fit in the 70% training sample, and held-out participants were assigned to the nearest cluster centroid. The top row shows mean standardized digital cognitive feature profiles for held-out participants assigned to each cluster. The bottom row shows longitudinal PACC trajectories for the same held-out participants. Across splits, the held-out cluster structure was reproducible, and participants assigned to Cluster 3 consistently showed the steepest PACC decline.

Supplementary Figure 5. Clinical progression on the CDR scale across cognitive phenotypes. A) Kaplan–Meier survival curves showing progression on the CDR scale over 240 weeks for Aβ negative participants and the four digital cognitive phenotypes identified among Aβ positive individuals. B) Hazard ratios for time to CDR progression for each phenotype relative to the Aβ negative reference group, with points representing estimated hazard ratios and horizontal bars indicating 95 percent confidence intervals. Cluster 3 shows the highest risk of clinical progression.

Supplementary Figure 6. Adjusted survival analyses for cognitive decline and CDR progression by digital cognitive cluster. Kaplan–Meier curves show time to meaningful PACC decline (A) and CDR progression (C) for Aβ− participants and Aβ+ cognitive clusters. Forest plots show adjusted hazard ratios for PACC decline (B) and CDR progression (D), with Aβ− participants as the reference group. Cox models were adjusted for age, sex, education, APOE ε4 carriership, and treatment assignment.

Supplementary Figure 7. Participant-level baseline and longitudinal tau PET maps across digital cognitive clusters. Paired tau PET maps are shown for each participant with available baseline and follow-up tau PET, organized by digital cognitive cluster. Within each cluster, each row represents one participant, with baseline tau PET shown on the left and longitudinal follow-up tau PET shown on the right. Warmer colors indicate higher tau PET signal.

Supplementary Figure 8. Proportion of individuals tau positive at different cutoff scores for both MTL and Neocortical Tau.
